# MiR-1-3p Suppresses Colorectal Cancer Cell Proliferation and Metastasis by Inhibiting YWHAZ-Mediated Epithelial–Mesenchymal Transition

**DOI:** 10.3389/fonc.2021.634596

**Published:** 2021-02-26

**Authors:** Guanghong Du, Xuelian Yu, Yun Chen, Wangting Cai

**Affiliations:** ^1^ Department of Geriatrics, Sichuan Provincial People’s Hospital, University of Electronic Science and Technology of China, Chengdu, China; ^2^ Organ transplant center, Sichuan Provincial People's Hospital, University of Electronic Science and Technology of China, Chengdu, China

**Keywords:** colorectal cancer, miR-1-3p, YWHAZ, epithelial–mesenchymal transition, progression

## Abstract

**Background:**

Colorectal cancer (CRC) is regarded as one of the most common malignancies in the world. MiR-1-3p was reported to be a tumor suppressor in CRC. However, the mechanisms have not been fully elucidated.

**Methods:**

To identify CRC-associated miRNA, microarray data set GSE30454 was downloaded from the Gene Expression Omnibus database (GEO), and miR-1-3p was screened out as a candidate. The expression of miR-1-3p was detected using quantitative real-time polymerase chain reaction (qRT-PCR) in CRC cell lines and tissues. CCK-8 assay and transwell invasion assay were performed to determine CRC cell line proliferation and invasion, respectively. The levels of YWHAZ and EMT-associated proteins were detected using western blotting.

**Results:**

Bioinformatic analysis showed that miR-1-3p was downregulated in CRC tissues, which is verified by our experimental validation. The overexpression of miR-1-3p significantly suppressed CRC cell proliferation and invasion. Further studies showed that YWHAZ was a direct target of miR-1-3p and mediated epithelial–mesenchymal transition (EMT) modulated by miR-1-3p.

**Conclusion:**

Our results demonstrated that miR-1-3p suppresses colorectal cancer cell proliferation and metastasis through regulating YWHAZ-mediated EMT, which may support a novel therapeutic strategy for CRC patients.

## Introduction

Colorectal cancer (CRC) is one of the most prevalent and fatal malignancies worldwide. There are over 1.8 million new CRC cases and 881,000 deaths that occurred in 2018 (1). In China, the incidence of CRC has been gradually increasing in recent years (2). Despite the growing body of treatments for CRC, the overall survival of patients with CRC is still far from satisfactory. Hence, identifying more molecules in CRC progression to support specific targeted drugs is important and urgent.

MicroRNAs (miRNAs) are a class of small non-coding RNA molecules of 20–22 nucleotides, which can directly bind to the 3′-untranslated region (3′-UTR) of target gene mRNAs to inhibit their translation (3). Increasing studies have reported that patients with tumor exist in a variety of disorders and abnormal expression of miRNAs. It is demonstrated that the dysregulated expression of several miRNAs, such as miR-124 (4), miR-625 (5) and miR-133a (6) contributes to the development and progression of CRC. Besides the miRNAs mentioned above, miR-1-3p (previously named miR-1) is identified as a tumor suppressor in various analyses of microRNA expression profiling (7–14). Recently, it is demonstrated that miR-1-3p could inhibit metastasis of CRC by restraining epithelial–mesenchymal transition (EMT) (15), but the underlying mechanisms still have not been fully elucidated.

In our study, the expression of miR-1-3p was detected in human CRC tissues and cell lines. Gain- or loss-of-function assays showed that miR-1-3p suppresses the development and progression of CRC. Furthermore, we found that an EMT-associated protein, YWHAZ, was a target of miR-1-3p. MiR-1-3p played its suppressive function in CRC through reversing YWHAZ-derived EMT.

## Materials and Methods

### Microarray Data

The miRNA profiling (GSE30454), containing 65 CRC samples and nine normal colonic mucosa samples, was downloaded from the GEO database for identifying the differentially expressed miRNAs (DEMs). The miRNA profiling, GSE38389 (68 tumors and 70 normal mucosa), GSE10259 (58 tumors and eight normal mucosa), GSE41655 (92 tumors and 15 normal mucosa) and GSE35982 (eight colorectal cancer tissues and their corresponding adjacent normal tissues), were also downloaded as test database.

### Identification of DEMs

GEO2R (http://www.ncbi.nlm.nih.gov/geo/geo2r/), a free online tool for identifying the DEMs from GEO database (16), was applied to detect the DEMs between CRC samples and normal colonic mucosa samples. Adjust *P*-value <0.01, combined with |log_2_FC| ≥1 was set as the thresholds for identifying DEMs.

### Patients and Specimens

A total of 20 patients with CRC in the Sichuan Provincial People’s Hospital were selected, and CRC tissues and adjacent normal tissues were obtained through surgery. All the patients did not receive preoperative chemotherapy. This study was approved by the Ethics Committee of the Sichuan Provincial People’s Hospital and all patients’ written informed consents were obtained.

### Cell Culture and miRNA Transfection

Human CRC cell lines (HT29, HCT116, SW480) were selected as test cells. All cells (American Type Culture Collection, ATCC) were cultured in RPMI 1640 (Hyclone) supplemented with 10% fetal bovine serum (FBS) (Gibco, Invitrogen) at a humidity of 5% CO2 at 37°C.

MiRNAs and expression plasmids were transfected into cells by Lipofectamine 2000 reagent (Invitrogen) according to the manufacturer’s instructions. The miR-1-3p mimic (HmiR0426), a non-specific miR control (HmiR0426-MR04), anti-miR-1-3p (miR-1-3p inhibitor, HmiR-AN0044), and a non-specific anti-miR control (CmiR-AN0001-AM01) were all purchased from FulenGen (Guangzhou, China).

### RNA Isolation and qRT-PCR

Total RNA was extracted using Trizol reagent (Invitrogen) and reverse transcribed for quantification using an NCode miRNA First-Strand cDNA Synthesis kit (Invitrogen). mRNA level of YWHAZ was measured as previously described (17). qRT-PCR was carried out using an SYBR Green PCR master mix (Applied Biosystems) on an ABI 7500HT system. The 2^−ΔΔCt^ method was used to measure the relative expression of miR-1-3p and YWHAZ. Each PCR amplification was performed in triplicate to verify the results. GAPDH was used as an internal reference of mRNA, U6 was used as an internal reference of miRNA. The primers for miR-1-3p (HmiRQP0044) and U6 (HmiRQP9001) were purchased from FulenGen (Guangzhou, China). The other primers for the target genes in the study are listed below.

YWHAZ: forward: 5′-ACTTTTGGTACATTGTGGCTTCAA-3′; reverse: 5′-CCGCCAGGACAAACCAGTAT-3′;

GAPDH: forward: 5′-TGCTTCAGGGTTTCATCCAG-3′; reverse: 5′- GACACTCGCTCAGCTTCTTG3-3′.

### CCK-8 Assay

Cells (5 × 10^3^) were seeded in 96-well plates in 100 μl medium. After treatment, cell viability was measured using the Cell Counting Kit-8 (CCK-8) kit (Beyotime) according to the manufacturer’s instructions. The extent of proliferation was evaluated every 24 h for 6 days.

### Transwell Invasion Assays

The invasive capacity of cells was performed using transwell precoated with matrigel (BD Biosciences). After incubation for 24 h, the cells that did not invade through the pores were carefully wiped away using a cotton-tipped swab, and the invaded cells were fixed with 4% paraformaldehyde, and then stained with Giemsa solution. Ultimately, five fields were randomly selected to count the cell number under an inverted microscope (Olympus).

### Prediction of Targets and Luciferase Reporter Assay

ENCORI (http://starbase.sysu.edu.cn/index.php) (18) and miRTarBase (http://mirtarbase.cuhk.edu.cn/php/index.php) (19) are online software programs that were used for miRNA target prediction.

The wild-type (wt) 3′-untranslated region (3′-UTR) fragment of YWHAZ that can bind to miR-1-3p or the mutant 3′-UTR fragments were synthesize by Sangon, China. Then, the segments were cloned into pMIRREPORT (Ambion) with firefly luciferase. Cells treated with control, miR-1-3p mimics, or miR-1-3p inhibitors were co-transfected with wild-type or mutants of YWHAZ 3′-UTR luciferase reporters together with Renilla plasmid. The dual-luciferase reporter assay system (Promega) was used to measure the luciferase activity in CRC cells after transfection for 48 h.

### Western Blot Analysis

Protein from cells was extracted using RIPA lysis buffer (Beyotime) with freshly added PMSF (Beyotime). The protein concentration was quantified using the Bradford method. All the antibodies used in our study were bought from Santa Cruz Biotechnology (Santa Cruz).

### Statistical Analysis

Data were analyzed using SPSS version 13.0 software. The Student t-test and the one-way ANOVA test were carried out for qRT-PCR and CCK-8 analyses. The correlation between miR-1-3p and YWHAZ was determined using the Spearman rank correlation test. A *P* value <0.05 was considered significant.

## Results

### MiR-1-3p Was Downregulated in CRC Tissues and Cell Lines

**Figure 1:**
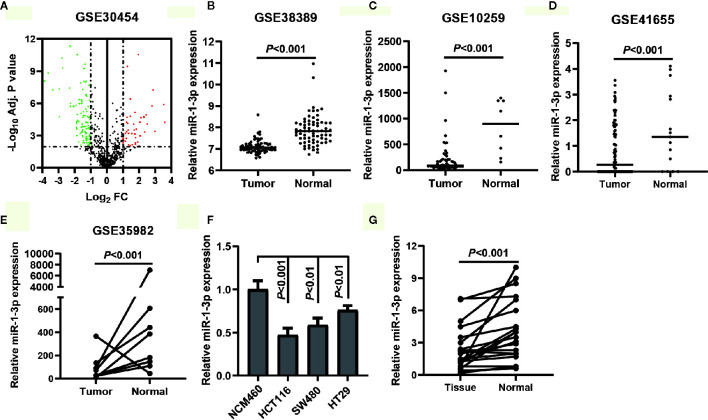
MiR-1-3p was downregulated in CRC tissues and cell lines. **(A)** Volcano plot of the DEMs (adjust *P*-value <0.01 and |logFC| ≥1 were set as the cut-off criteria). **(B–E)** The expression of miR-1-3p from the GEO database GSE38389, GSE10259, GSE41655 and GSE35982. **(F)** The expression of miR-1-3p in three CRC cell lines (HCT116, SW480, HT29) was compared with that in control. **(G)** The expression of miR-1-3p in tumor tissues was compared with the normal control.

To identify the DEMs of miRNA array GSE30454 downloaded from GEO, we conducted a differential expression analysis using GEO2R. The results showed that a total of 149 DEGs were identified, including 105 downregulated and 44 upregulated DEMs (**Figure 1A**). The top five downregulated and upregulated DEMs were listed in **Table 1**. Since the top one downregulated DEM, miR-129-5p, was well studied in CRC (20, 21), miR-1-3p was selected for the further study. To verify the expression of miR-1-3p in CRC, four miRNA expression profiling, GSE38389, GSE10259, GSE41655 and GSE35982 were downloaded and analyzed. It was demonstrated that miR-1-3p was significantly downregulated in CRC (**Figures 1B–E**). Additionally, the expression of miR-1-3p in CRC cell lines and our collected CRC tissue samples were also detected. As shown in **Figures 1F, G**, miR-1-3p was downregulated in HCT116, SW480, HT29 and CRC tissues, compared to the normal control NCM460.

**Table 1:** Top 10 DEMs between CRC tissues and normal colonic mucosa samples from the miRNA profiling GSE30454.

miRNA	logFC	t	B	P.Value	adj.P.Val
**Down-regulated**					
hsa-miR-129-5p	−2.77804	−10.55171	20.4586	2.03E-13	5.81E-11
hsa-miR-1-3p	−2.74918	−9.63754	17.7827	3.11E-12	5.09E-10
hsa-miR-137-3p	−2.27167	−11.9235	24.2607	4.12E-15	4.72E-12
hsa-miR-488-3p	−1.82694	−10.89459	21.4332	7.49E-14	2.86E-11
hsa-miR-153-3p	−1.34732	−10.0189	18.9125	9.84E-13	2.25E-10
**Up-regulated**					
hsa-miR-1273a	3.53691	6.62772	8.3016	4.82E-08	1.31E-06
hsa-miR-31-5p	2.86879	7.72831	11.8621	1.29E-09	5.67E-08
hsa-miR-663b	1.46511	7.08333	9.7834	1.07E-08	3.94E-07
hsa-miR-183-5p	1.96749	11.03949	21.8401	4.94E-14	2.83E-11
hsa-miR-1826	1.27283	9.7969	18.2571	1.92E-12	3.66E-10

t, t-statistic; B, B-statistic.

These results prompt us to investigate the potential role of miR-1-3p in CRC suppression.

### MiR-1-3p Suppressed CRC Cell Proliferation and Invasion

To explore the biological function of miR-1-3p in CRC, gain- and loss-of-function assays were performed.

**Figure 2:**
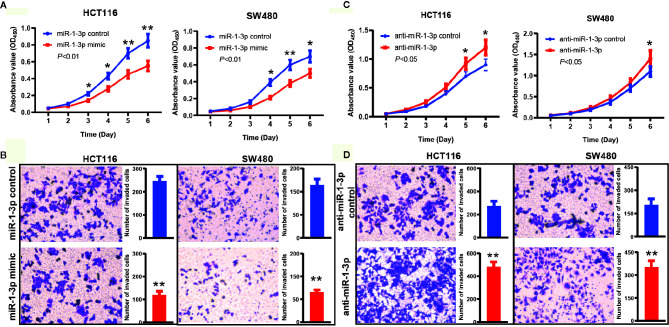
MiR-1-3p suppressed CRC cell proliferation and invasion. **(A)** The effect of miR-1-3p on cell proliferation was evaluated by CCK-8 assay after miR-1-3p transfection of HCT116 and SW480 cells. **(B)** Data of transwell assay for HCT116 and SW480 cells. The cells were counted under a microscope in five randomly selected fields. **(C)** The effect of anti-miR-1-3p on cell proliferation was evaluated by CCK-8 assay after anti-miR-1-3p transfection of HCT116 and SW480 cells. **(D)** Data of transwell assay for HCT116 and SW480 cells. The cells were counted under a microscope in five randomly selected fields. *P < 0.05, **P < 0.01.

Firstly, we transfected miR-1-3p mimic into HCT116 and SW480 cells to investigate the role of miR-1-3p, respectively. The CCK-8 assays results showed that miR-1-3p could remarkably inhibit the proliferative ability of HCT116 and SW480 cells (**Figure 2A**). In addition, a transwell invasion assay was employed to measure the effects of miR-1-3p on CRC invasion. The results demonstrated that upregulation of miR-1-3p significantly decreased the cell number of invaded HCT116 and SW480 (**Figure 2B**).

Furthermore, we also transfected anti-miR-1-3p into HCT116 and SW480 cells to decrease the expression of miR-1-3p. On the contrary with the above results, we found that the proliferation activity of HCT116 and SW480 cells in the anti-miR-1-3p group were significantly higher than those in the control group (**Figure 2C**). Transwell invasion assays showed that downregulated miR-1-3p expression significantly increased the cell invasion capabilities of CRC cells compared to the control group (**Figure 2D**).

These findings demonstrated the role of miR-1-3p in inhibiting aggressive phenotype of CRC cells.

### Prediction and Validation of YWHAZ as a Direct Target of miR-1-3p

**Figure 3:**
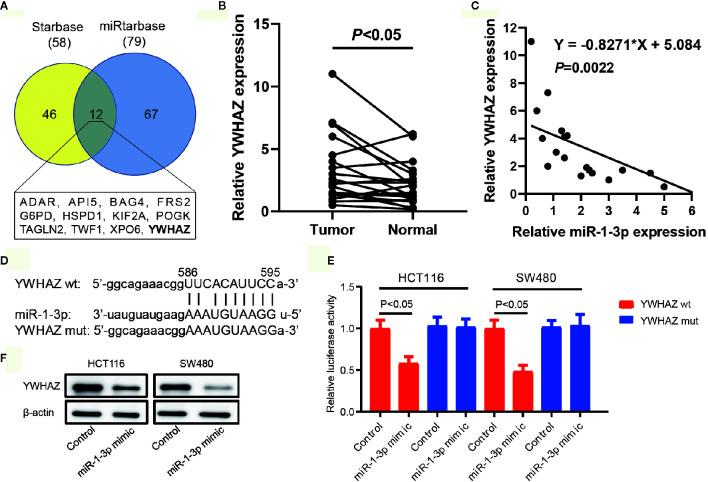
Prediction and validation of YWHAZ as a direct target of miR-1-3p. **(A)** Venn diagram of the overlapping target genes using the Starbase and miRtarbase databases. The 12 overlapping target genes were listed. **(B)** The expression of YWHAZ in our collected CRC tissues and adjacent normal tissues. **(C)** The expression of miR-1-3p and YWHAZ mRNA was detected in CRC tissues by qRT-PCR analysis. A statistically significant inverse correlation between miR-1-3p and YWHAZ mRNA was observed in CRC specimens. **(D)** Diagram of YWHAZ 3′UTR containing 1 putative conserved target sites for miR-1-3p, which were identified using the Starbase and miRtarbase databases. **(E)** Results of luciferase reporter assays in HCT116 and SW480 cells, with co-transfection of wt or mt 3′UTR and miR mimic and inhibitor, as indicated. **(F)** The protein expression of YWHAZ in HCT116 and SW480 cells transfected with miR-1-3p.

It is acknowledged that miRNA exerts its function *via* regulating the expression of its target gene. To predict the potential targets of miR-1-3p, publicly available algorithms (ENCORI and miRtarbase) were used in our study. By ENCORI, 58 potential target genes were predicted with the screening criteria of programNum ≥6 and pan-Cancer ≥5. Meanwhile, 79 potential target genes were predicted by miRtarbase with high confidence. 12 overlapping potential target genes were screened out and listed in **Figure 3A**. Among these genes, YWHAZ, a central hub protein for EMT, was selected for further experimental verification. On the contrary with the expression of miR-1-3p in CRC, we found that YWHAZ were upregulated in CRC tissues compared to adjacent normal tissues (**Figure 3B**). Furthermore, the expression levels of YWHAZ and miR-1-3p exhibited a significant inverse correlation in 10 CRC samples (r = −0.8271, *P* = 0.0022, **Figure 3C**). These results supported our hypothesis that YWHAZ was a target gene of miR-1-3p.

The predicted site in YWHAZ 3′-UTR that can be bound by miR-1-3p is illustrated in **Figure 3D**. We consequently explored whether YWHAZ was a direct target of miR-1-3p in CRC cells. The luciferase reporter assay showed that miR-1-3p mimic significantly inhibited the luciferase activity in HCT116 and SW480 cells with wt-YWHAZ-3′ UTR vector, but not in mutant YWHAZ-3′ UTR (**Figure 3E**). We also analyzed the expression of YWHAZ in GSE29760, which was an expression profiling in HCT116 cells transfected with miR-1-3p precursor molecule. We found that the expression of YWHAZ was decreased in miR-1-3p-transfected HCT116 cells (**Table S1**). Moreover, transfected miR-1-3p mimic resulted in significant reduction of YWHAZ protein (**Figure 3F**).

These results indicated that YWHAZ was a direct target gene of miR-1-3p.

### MiR-1-3p Modulated EMT in an YWHAZ-Dependent Way

**Figure 4:**
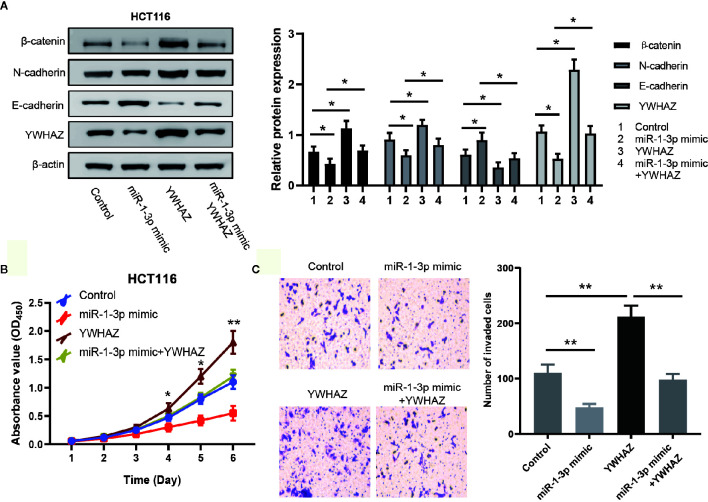
MiR-1-3p modulated EMT in an YWHAZ-dependent way. **(A)** Western blotting of EMT markers and YWHAZ were performed to determine whether miR-1-3p mediated EMT in a YWHAZ-dependent way. **(B)** The effect of miR-1-3p and YWHAZ on cell proliferation was evaluated by CCK-8. **(C)** Results of transwell assay, which was carried out to evaluate the effect of cell invasion after transfection are shown. *P < 0.05, **P < 0.01.

EMT is a critical process during tumor metastasis by which epithelial cells lose their cell–cell adhesion and obtain mesenchymal features (22). Combining the results above and the previously report of YWHAZ involved in EMT, we hypothesized that miR-1-3p modulated EMT in CRC cells through regulating the expression of YWHAZ. To assess this hypothesis, YWHAZ expression vector was transfected into miR-1-3p-overexpressing HCT116 cells. The expression of YWHAZ and known molecular markers of EMT (N-cadherin, E-cadherin, *β*-catenin) was examined by western blotting. The results showed that miR-1-3p decreased the expression of YWHAZ with the downregulated expression of N-cadherin, *β*-catenin and upregulated expression of E-cadherin, which was neutralized by the overexpression of YWHAZ (**Figure 4A**). And then overexpression of YWHAZ reduced the inhibition of proliferation and invasion ability by miR-1-3p in HCT116 cells (**Figures 4B, C**).

These results together indicate that miR-1-3p modulates EMT in an YWHAZ-dependent way.

## Discussion

Colorectal cancer (CRC) is a high heterogeneous and fatal malignancy in the world. Despite major advances in diagnostics and treatment, the complex pathogenesis of CRC has no satisfying therapies and prognosis (14). Therefore, in-depth exploration of the pathogenesis of CRC will be crucial for seeking and developing a promising therapeutic target to improve the survival of patients with this tumor.

Currently, miRNAs have been regarded as popular tumor molecular markers which possess a variety of biological functions. MiR-1-3p was first reported to involve in cardiac morphogenesis and cell cycle (23). Previous research on the epigenetics of CRC showed that miR-1-3p was silenced in association with CGI methylation in HCT116 cells, suggesting its role of tumor suppressor (8). With in-depth study, the tumor suppressor role of miR−1-3p has been proven (24, 25). However, the mechanism of miR-1-3p in the development of CRC has not clarified. Public data and our data demonstrated that the expression of miR-1-3p was decreased in CRC tissue and cell lines with increased malignant behavior of CRC. Functional experiments demonstrated that CRC cell lines with the miR-1-3p mimic showed lower cell proliferation and invasion capacity.

To explore the underlying mechanism of tumor suppression by miR-1-3p, YWHAZ was identified as a direct target gene of miR-1-3p in CRC cell lines. The trend of YWHAZ mRNA was contrary to miR-1-3p, indicating that the biological effect of miR-1-3p may be mediated by inhibiting the YWHAZ-involved pathway. YWHAZ (also named 14-3-3ζ) is reported to be a central hub protein in tumor progression (26). The oncogene function of YWHAZ has been well recognized in multiple types of cancers by participating in cell growth, cell cycle, apoptosis, migration, and invasion (17, 27). It is observed that the expression of YWHAZ is increased in 46 colorectal cancer (CRC) tissues (28). Additionally, YWHAZ silencing significantly decreased colony formation in CRC cells, suggesting its role in conferring malignant phenotype *via* extracellular vesicles (29). A recent study demonstrated that YWHAZ modulates EMT in CRC by interacting with thyroid hormone receptor interactor 13 (TRIP13) (30). So, we hypothesize that miR-1-3p plays its suppressive role in CRC by inhibiting YWHAZ-mediated EMT. As expected, we found that overexpression of YWHAZ neutralized the downregulation effect of EMT caused by miR-1-3p, supporting our previous hypothesis that miR-1-3p suppresses CRC cell proliferation and metastasis by inhibiting YWHAZ-mediated EMT. In follow-up studies, we will further verify the results of the study *in vivo* through animal model tests.

## Conclusion

Our comprehensive research indicates that miR-1-3p was significantly downregulated in CRC tissues and cell lines, and YWHAZ is a direct target gene of miR-1-3p. MiR-1-3p can bind to the 3′URT of YWHAZ mRNA, thereby inhibiting YWHAZ-mediated EMT. These findings indicate a novel mechanism of tumor suppression by miR-1-3p, supporting a novel therapeutic strategy for CRC patients.

## Data Availability Statement

The raw data supporting the conclusions of this article will be made available by the authors, without undue reservation.

## Ethics Statement

The studies involving human participants were reviewed and approved by the Ethics Committee of the Sichuan Provincial People’s Hospital. The patients/participants provided their written informed consent to participate in this study.

## Author Contributions

WC conceived and supervised the study. WC and GD designed experiments. GD performed experiments. GD, XY, and YC analyzed data. WC and GD wrote the manuscript. All authors contributed to the article and approved the submitted version.

## Funding

The present study was supported by the Chinese National Natural Science Foundation (81502075).

## Conflict of Interest

The authors declare that the research was conducted in the absence of any commercial or financial relationships that could be construed as a potential conflict of interest.

## Supplementary Material

Supplementary Table 1 The expression data from GSE29760.
